# Oritavancin in LVAD related infections - a chance for shortening therapy and improving outcomes

**DOI:** 10.1186/s12879-025-11692-x

**Published:** 2025-10-21

**Authors:** Marta Załęska-Kocięcka, Szymon Mielczarek, Piotr Góral, Kamil Marcinkiewicz, Katarzyna Byczkowska, Karina Zatorska, Justyna Cieślik, Karolina Paszyń, Aleksandra Tomaszek, Piotr Kołsut, Katarzyna Jóźwik-Plebanek, Jarosław Kuriata, Jarosław Szymański, Przemysław Leszek

**Affiliations:** 1 Department of Heart Failure and Transplantation, Mechanical Circulatory and Heart Transplantation Unit , Cardinal Wyszynski National Institute of Cardiology, Warsaw, Poland; 2 Department of Valvular Heart Disease, Cardinal Wyszynski National Institute of Cardiology, Warsaw, Poland; 3 Department of Microbiology, Cardinal Wyszynski National Institute of Cardiology, Warsaw, Poland; 4 Department of Cardiac Surgery and Transplantation, Cardinal Wyszynski National Institute of Cardiology, Warsaw, Poland; 5 Teaching Department of Anesthesiology and Intensive Care, Faculty of Health Sciences, Medical University of Warsaw, Warsaw, Poland; 6 Department of Endocrinology and Radioisotope Therapy, Miliary Institute of Medicine - National Research Institute, Warsaw, Poland

**Keywords:** LVAD infections; drive-line infections; oritavancin

## Abstract

**Background:**

LVAD utility in heart failure patients is growing. Despite advancement in technology rates of LVAD- related infections remain high limiting outcomes. We aimed to assess the effectiveness and safety of oritavancin for LVAD-related Gram-positive infections.

**Methods:**

A retrospective study evaluating adult LVAD patients who received ≥ 1 oritavancin dose for treatment of Gram-positive LVAD-related infection between September 2022 and December 2024. Oritavancin was given at the end of shortened standard antibiotic therapy. The primary endpoint was 90-day clinical and microbiological cure of the primary or relapsing infection within 90 days from the first oritavancin dose. Secondary endpoints were adverse events.

**Results:**

Overall, nine patients were included. All were male, mean age was 53 ± 9 years. In total they experienced 10 LVAD- related episodes. Most episodes (80%) were deep drive-line infections. The organism most commonly responsible for infection was *Staphyloccocus aureus*. Oritavancin was administered after a median time of 31 ± 13 days of standard antibiotic therapy. In total 18 doses of oritavancin were administered with a median of 2 per case separated on average by 20 ± 4 days. Primary endpoint was achieved in 90% of cases, shortening therapy by a median time of 24 days. Serious adverse events occurred in two of 9 patients: delirium and shivers, both with transient increase of inflammatory markers.

**Conclusion:**

Oritavancin has a potential to shorten inhospital therapy and improve outcomes in LVAD-related Gram-positive infections in advanced heart failure patients. The study is the first to report delirium and transient increase in inflammatory biomarkes as side effects.

## Introduction

Due to heart failure pandemic left ventricle assist device (LVAD) utility is growing. It significantly improves survival and quality of life in advanced heart failure patients with reduced ejection fraction. Despite remarkable improvement in survival since introduction of the third generation centrifugal continuous-flow pumps LVAD-related infections and particularly drive-line infection (DLI) rates remain high thereby limiting therapy. According to INTERMACS REGISTRY nearly 40% of patients experience at least one episode of LVAD-related infection during 5 years of support [1]. Data on reoccurrence are scarce and no definition of DLI relapse or reinfection exists. In fact, biofilm-forming bacteria along with foreign body are ideal conditions for chronic infection with episodes of exacerbation and remission. Data on preventive measures focusing on wound care strategies are limited. It is important to acknowledge that while driveline infections are microbiological in nature, mechanical factors play a significant contributory role in their development and persistence. Younger, more physically active patients may be particularly vulnerable due to repetitive mechanical stress at the driveline exit site, which can lead to skin breakdown and chronic inflammation, thereby predisposing to infection. In fact the main trigger is recurrent driveline exit site trauma due to accidental tension, which creates a conduit for entry of pathogens [2]. Gram positive bacteria are the most frequent pathogens with Staphylococcus aureus being the majority (up to 60% of DLI infections) [3]. Thus, in terms of etiology and pathophysiology, DLI share features of surgical site infection. However, involvement of prosthetic material exhibits a biofilm-friendly environment and treatment challenges include long term treatment consistent to infective endocarditis scheme. According to the International Society of Heart and Lung Transplantation (ISHLT) guidelines a minimum of 2-week therapy is indicated for superficial DLI and 6–8 weeks in case of deep-seated DLI [4]. This poses a great logistic and economic burden mainly attributable to prolonged hospital stay. Long-acting antibiotics like oritavancin enable single or multiple dose treatment with out-of-hospital stay in-between doses potentially limiting costs, resources use and hospital- acquired complications. Oritavancin was approved by Food and Drug Administration and European Medicines Agency for clinical use in 2014 with a singular on-label indication: acute bacterial skin and skin structure infections [5]. It is a lipoglycopeptide bactericidal agent with the exceptionally long half-life of 245 h. It exhibits potent activity against Staphylococcus aureus including methicillin-susceptible (MSSA) and methicillin-resistant (MRSA), vancomycin-intermediate resistant (VISA), vancomycin resistant (VRSA) strains, Enterococcus spp.including vancomycin resistant enterococci (VRE), and Streptococcus spp [6]. Complex mechanisms of action ensures a rapid bactericidal effect and high anti- biofilm activity [4]. The latter could be crucial in case of DLI due to prosthetic material involvement along with Staphylococcus aureus a potent biofilm former. What is more oritavancin displayed potent and stable activity (MIC90 range of 0.06 to 0.5 mg/L) over a 10-year period (2010 to 2019) against those pathogens [7]. Given oritavancin’s long elimination half-time of over 12 days consecutive doses might be given 2 weeks apart potentially covering long term treatment without a need of hospitalization. Such properties make oritavancin a reasonable alternative for DLI in LVAD patients. Therefore, this study aimed to assess the effectiveness and safety of oritavancin as additional therapy for DLI due to Gram-positive organisms.

## Methods and methods

This is a retrospective, observational cohort study evaluating adult LVAD patients hospitalized between 2022 and 2024 in a tertiary center. Out of 90 patients under center supervision subjects who received at least one dose of oritavancin for the sequential treatment of DLI or other LVAD related infections to shorten therapy were included.

This study was approved by Local Ethics Comity (No 103537) by Stefan Cardinal Wyszynski National Institute of Cardiology in Warsaw, Poland. Due to retrospective nature of the study informed patient consent was waived by Local Ethics Comity of Stefan Cardinal Wyszynski National Institute of Cardiology in Warsaw, Poland. The study adhered to the Declaration of Helsinki. Data extracted from the electronic health recorder (ER; Clininet) as well National Registry of Mechanical Circulatory Support included patient demographics, comorbidities, infection and treatment information, length of stay, adverse events, and outcomes, including hospital readmission, re-infection, and mortality. The index organism(s) was the Gram-positive organism(s) referred by a treating physician as the etiology of the LVAD-related infection. Infections in which more than one pathogenic organism was identified were classified as polymicrobial. DLI and other LVAD-related infections were classified according to Kormos et al. [8]. Additionally, classification was applied in terms of nature of onset with three categories: (1) primary infection, (2) relapsing infection, and (3) treatment failure. In case of primary infection oritavancin was given after few weeks of standard antibiotic therapy to shorten hospital stay. Infection relapse was defined as reoccurrence of infection of the same etiology within 90 days after last dose of standard antibiotic course in previous hospital stay. Treatment failure was defined as presence of signs, symptoms, laboratory and/or imaging results suggesting presence of active infection despite standard antibiotic therapy in the index hospital stay. Acute kidney injury (AKI) was defined as meeting at least stage I injury in line with recommendation by Kormos [8]. The primary endpoint was 90-day clinical and microbiological cure of infection from the first oritavancin dose. The clinically evaluable patient population included those with follow-up within the healthcare system at 90 days. Adjudication of effectiveness outcomes was performed by a multidisciplinary team.

The secondary endpoint was the incidence of adverse events. These included acute kidney injury andf incidence of infusion-related reactions or anaphylaxis. An adverse event was classified as serious if fatality resulted requiring prolonged hospital stay or rehospitalization.

## Results

There were 12 LVAD patients treated with oritavancin in years 2022–2024 in our center that in total experienced 13 infection episodes. Three episodes in three patients were excluded from the analysis due to non-LVAD related infections.

All 9 patients experienced 10 episodes treated with targeted oritavancin therapy were men with a mean age of 53 ± 9 years. All were supported with LVAD as a bridge to transplantation. Patient characteristics are displayed in Table 1.

**Table 1:** Patients baseline characteristics

Baseline characteristics	*n* = 9
Age, *years*, mean ± SD	53 ± 9
Male sex *n*,* (%)*	9 (100%)
HF of ischemic etiology n, (%)	5 (55%)
NYHA class	
I	3 (33%)
II	5 (55%)
III	1 (11%)
IV	0
Duration on LVAD support,*days* mean ± SD	660 ± 560
LVAD type	
HeatMate III	8 (88%)
Diabete mellitus n, (%)	6 (66%)
COPD n, (%)	1 (11%)
Arterial Hypertension n, (%)	4 (44%)
Ventricula arrhytmias n, (%)	6 (66%)
Atrial fibrillation n, (%)	5 (55%)
CKD n, (%)	5 (55%)
creatinine level on the day of oritavancin infusion [mg%], mean ± SD	1,15 ± 0,22
Pulmonary hypertension	8 (88%)
CIED	8 (88%)

*CIED* Cardiovascular implantable electronical device, *CKD* Chronic kidney disease,* HF* Heart failure, *COPD* Chronic obstructive pulmonary disease,* NYHA* New York Heart Association

Among 10 episodes (no VII and VIII in one patient) including one reoccurence of LVAD-related infections, all appeared late (> 90 days) following LVAD implantation. The mean time from LVAD placement to index infection was 660 days (standard deviation 630 days). The majority (8 out of 10) were deep drive-line infections (DLI) and the remaining two other LVAD-related infections (Table 2). The most common etiology was MSSA (5 out of 10). Three episodes were polymicrobial (3 out of 10), two of which were accompanied with bloodstream infections: one with the index pathogen (episode I), other with another gram-positive bacteria (episode VI) (Table 2). The total number of doses administered was 18 with median of 2 per patient (min. 1; max. 3). Three patients received also maintaining oral antibiotics when discharged home after the first oritavancin dose (Table 2). And one patient prophylactic extension (Table 2).

**Table 2:** Infection and treatment details§

ID	Time (days) from LVAD implantation to infection onset	Type of LVAD-related infection	Primary pathogen	Infection type	Concomitant infection during the index hospitalization	Number of hospitalizations due to infection before index stay	Duration of oral outpatient abx before t he index hospitalization	Duration of the index hospital stay	Targeted abx for primary pathogen	Surgical debridement	Number of oritavancin doses	Time of consequtive doses; (DX-numember of dose Xd -number of days from the first dose)	Maintainance oral abx after first oritavancine dose	OHT	Time (days) from the first oritavancine dose to OHT	Outcome
I	714	SSI (minitoracothomy)	*E. faecalis*	relapse	*E. faecalis*: urinary and BSI *S. hominis*: BSI *K. pneumoniae* ESBL: LVAD-related infection	3	0	48 (43) days	vancomycin, meropenem	in previous and index hospital stay	3	D1-0d; D2-21d; D3-44d	no	yes	142 days	cure
II	506	deep DLI	*Corynebacterium* spp.	primary	no	0	NA	42 (41) days	vancomycin	in the index hospital stay	1	D1-0d, D2-21d	NA	no	NA	cure
III	639	deep DLI	*S. aureus* (MSSA)	primary	no	0	NA	36 (27) days	cloxacillin, rifampicin, gentamycin	in the index hospital stay	2	D1-0d, D2-21	2 weeks prophylaxis with TMP-SMX	yes	30 days	cure
IV	199	deep DLI	*S. aureus* (MSSA)	primary	no	0	NA	33 (33) days	cloxacillin, rifampicin	no	1	D1-0d	3 -week treatment: cloxacililin and rifampicin	no	NA	cure
V	392	deep DLI	*S. aureus* (MSSA)	relapse	no	0 (only ambulatory treatment)	26	35 (35) days	cloxacillin, rifampicin, gentamycin	in the index hospital stay	2	D1-0d, D2-14d	4-week treatment: cloxacililin and rifampicin	yes	69 days	cure
VI	2088	deep DLI	*St. Capitis (MRCNS) Achromobacter xylosixidnas*,* Corynebacterium amycolatum.*	primary	*S. epidermids: BSI*	0	NA	56 (54) days	Piperacillin-tazobactam vancomycin	in the index hospital stay	1	D1-0	NA	yes	130 days	failure
VII^a^	149	deep DLI	*S. aureus* (MSSA)	relapse and failure of index standard treatment based on PET	no	1	14	5 (5) days	cloxacillin	no	2	D1-0d, D2-20d	no	no	NA	cure
VIII^a^	323	deep DLI	*S.aureus* (MSSA)	primary	no	0	NA	24 (24) days	cloxacillin, rifampicin	no	2	D1-0, D2-28d	6-week treatment: cloxacililin and rifampicin	no	NA	cure
IX	1162	deep DLI	*S. aureus* (MSSA)	relapse	1	1	14	31 (31) days	cloxacillin, rifampicin	no	2	D1-0d, D2-14d	no	no	NA	cure
X	94	SSI (sternotomy)	*E. faecium* (VRE); *S. epidermidis* (MRCNS)	relapse and failure of index standard treatment based on PET	no	1	30	22 (19) days	vancomycin	no	2	D1-0d, D2-24d	no	no	NA	cure

*Abx* Antibiotics, *OHT* Orthotopic heart transplantation, *D* -day, *NA* Not applicable, *TMP-SMX* Trimethoprim- sulfamethoxazole

^a^The episodes belong to the same patient

In half of episodes (5 out of 10) oritavancin was used for treatment of the primary infection and in the other half for relapsing cases after a median time of 32 (min 5 max 54) days of standard therapy, (Table 2).Among the latter, in two cases (episode VII, X) standard antibiotic treatment in the index hospitalization was unsuccessful based on Positron Emission Tomography (PET-CT) evaluation. Among 5 episodes of infection relapse, those were following a median number of 1,5 (min. 1; max. 3). hospitalizations before the index one. In half of cases surgical treatment was performed in the index hospitalization (episode I, II, III, V,VI) and in one additionally also in the previous treatment course (episode I).

The primary point was achieved in 90% of cases (in all episodes but ep. VI) (Table 2). The unsuccessful episode VI was polymicrobial and treated with only one dose of oritavancin due to occurrence of serious adverse event after the first dose. Oritavancin utilization resulted in shortening treatment scheme and subsequently hospitalization in 9 out of 10 episodes by a mean of 24 ± 12 days of hospital stay per patient. Four patients presenting four different episodes underwent heart transplantation after a median time of 92 ± 52 days (min.30; max 142 days). There was no relapse of the index pathogen infection in the early postoperative time in all but the one patient who did not meet the primary endpoint (Table 2). The remaining 5 patients after a mean time of 305 ± 148 days are still supported with LVAD.

The secondary end-point was achieved in two patients that in total received 18 oritavancin doses. In episode VI there was an incident of shivers during the first oritavancin infusion. The infusion was not terminated as the patient informed the medical team when the infusion ended. He reported shivers the day after. It was accompanied by marked increase of inflammatory markers in the morning the day after infusion. Laboratory tests revealed acute increase of CRP from 0,21 mg% to 4,29 mg%, procalcitonin 0,06 to 3.28 ng/ml and white blood cells from 5,7 with 62% neutrophils to 7.9 with 85% of neutrophils. There were no signs or symptoms of new ongoing infection, the parameters decreased gradually without additional intervention over 3 days. However, neither clinical signs and symptoms of infection were observed nor controlled microbiological tests were positive. In episode V delirium (agitation and confusion) occurred at the end of the second dose infusion. Infusion was ceased and delirium receded within 30 min and did not recur, and no other cause was identified. Delirium was also accompanied by transient albeit unremarkable increase of inflammatory markers CRP from 0,89 mg% to 1,8 mg%, procalcitonin 0,06 to 0,22 ng/ml and white blood cells from 5,9 with 73,8% neutrophils to 7.2 with 63,3% of neutrophils. In both cases increase of inflammatory markers was transient (with normalization within 2 days) with signs and symptoms of new infection.

## Discussion

The burden of heart failure continues to rise globally and consequently utilization of long term left ventricular support (LVAD). Given marked technologic advancements in device design and survival improvement indications have evolved. In recent years in the United States most patients undergo LVAD implantation as destination therapy mode. This translates into long-term support which is the main risk factor for LVAD-related infections. Additionally, these types of infections are not only frequent but also challenging to treat. The most common form that is drive-line infection (DLI). In terms of pathogens and tissue involvement they present as typical acute bacterial skin and skin structure infections (ABSSSI). On the other hand, involvement of foreign body and its continuum with mediastinum and finally vascular system gives the infection the highest priority comparable to infective endocarditis on prosthetic valves. This is reflected by treatment guidelines by the International Society for Heart and Lung Transplantation (ISHLT) that recommend oral or intravenous antibiotics for a minimum of 2 weeks, or until infection has resolved in cases of superficial DLI, whereas in cases of deep DLI minimum of 6–8 weeks of intravenous antibiotic therapy along with surgical control, often followed by a long-term (oral) antibiotic suppression therapy [4]. However, there is no consensus on the optimal antibiotics, and novel long-acting agents such as oritavancin have emerged as promising options. Oritavancin, originally approved for acute bacterial skin and skin-structure infections (ABSSSI), offers several advantages in this context: a broad Gram-positive spectrum (including Staphylococcus aureus and enterococci, an extensive volume of distribution with excellent tissue penetration, and an extremely long half-life permitting weekly dosing. These features allow for simplified therapy of LVAD-related infections with potentially fewer in-hospital days. Importantly, oritavancin’s multiple mechanisms of action (inhibition of cell-wall synthesis and disruption of bacterial membrane integrity) enable it to effectively kill biofilm-embedded, slow-growing staphylococci [9]. It was found that oritavancin sterilized biofilms of not only MSSA, but also MRSA and VRSA at minimal biofilm eradication concentrations [9].

Its advantages have already been evaluated in many other off label scenarios including blood stream infections, endocarditis and osteomyelitis, with encouraging results [5].

To our knowledge, this the first report of a cohort study evaluating use of oritavancin solely in LVAD -related infections. It was found that targeted treatment with oritavancin given after shortened standard antibiotic therapy led to successful treatment. The most common etiology was *Staphyloccocus aureus*, that is related up to 60% of DLIs as previously reported [3]. Oritavancin was given after a median time of 32 (min 5 max 54) days of standard therapy. Given ISHLT recommendation of antibiotic therapy duration of 42–56 days, its use allowed to shorten therapy and hospitalization on average by 24 days.

It is a subject of debate if use of antibiotics that are approved certainly for ABSSSIs may be considered ‘off-label’ when used for treating DLIs [10]. SOLO trials designed to compare single-dose intravenous oritavancin vs intravenous vancomycin in the treatment of ABSSSIs excluded infections involving prosthetic material [11, 12]. The findings of our study suggest application of oritavancin in the settings of LVAD-related infections. Yet, similar dilemma regards another glycopeptide dalabavancin that despite growing evidence of its efficacy in multiple “ off label” scenarios including LVAD-related [13]. Oritavancin shares many of dalbavancin’s advantages (weekly dosing, potent anti-staphylococcal activity) and additionally has activity against *Enterococcus* (including most vancomycin-resistant strains), which could be relevant in polymicrobial LVAD infections.

In line with Mansoor who evaluated dalbavancin we found that oritavancin is effective and allows reduction of length of stay (LOS) in LVAD- related infections [14]. The positive outcomes in our cohort mirror those seen with dalbavancin, reinforcing that oritavancin is a viable alternative for managing these difficult infections. Notably, our results and prior dalbavancin studies employed these agents after an initial course of conventional therapy, essentially as a switch or consolidation therapy.

While our results are promising, we do not propose oritavancin as a replacement for standard antibiotic therapy, particularly in deep-seated or complex driveline infections. Instead, its role may lie in adjunctive or consolidation therapy—after initial standard treatment—to facilitate outpatient management and reduce hospitalization, especially when indwelling catheters or OPAT services are not available or feasible. Future research should clarify the optimal timing of such a switch – for instance, whether earlier introduction of a long-acting agent could further improve convenience without compromising efficacy.

Consequently, Poliseno proved significant cost reduction with long acting glycopeptides use attributable to shorter LOS and indicated that financial benefits are most striking in scenarios requiring long term therapy such as cardiac implantable electronic device (CIED) infections, which can be easily translated into LVAD-related infections [13]. In the current study, LOS was shortened on average by 24 days. This means savings related to hospitalization length but also to indirect costs to health care such as burden of the indwelling pathogens in health-care settings. It is potentially a cost-effective alternative for outpatient parenteral antibiotic therapy (OPAT) service with frequent home visits, which remains unavailable in many countries including Poland. In addition, long-acting agents allow for avoidance of intravenous catheters, and related adverse outcomes such as bloodstream infection or deep vein thrombosis. Finally, shorted hospital stay means less muscle wasting which is a cornerstone of persisting weakness and fatigue negatively affecting the quality of life of already fragile advanced heart failure patients [15].

In terms of safety, the overall secondary endpoint was achieved in 20% (2 out of 10 episodes). Additionally, the events observed in our study were not previously reported in other studies. The first one, delirium, occurred at the end of infusion of the second dose repeated after 14 days. There were no side effects present during the infusion of the first dose in the subject. There were also no other factors that the delirium could be attributable to. To our knowledge, delirium has not been previously reported with oritavancin and is puzzling given the drug’s limited penetration across the blood-brain barrier. After drug discontinuation signs and symptoms resolved. There were no abnormalities computed tomography scan or any persistent neurologic deficits present.

The second event manifested with chills during infusion and abrupt growth of inflammatory markers the day after, reminiscent of an acute infusion-related reaction. No hypotension, rash, or organ dysfunction occurred, and inflammatory markers normalized within 3 days without additional therapy. We hypothesize that this transient inflammatory syndrome might have been triggered by rapid bacterial killing and release of cell components, given oritavancin’s potent bactericidal activity.

Notably, neither of these complications was observed in the large Phase 3 trials or prior case series – in the SOLO trials [11, 12]. They resolved spontaneously and did not result in long-term sequelae; these novel observations highlight the need for continued pharmacovigilance as oritavancin sees broader use in new patient populations.

Finally, it is worth noting that LVAD support requires therapeutic anticoagulation with vitamin K antagonist, whereas oritavancin may falsely elevate INR results. However, it is a result of in vitro interaction with phospholipids in certain laboratory tests not actual INR time prolongation. In fact such impact on measurements are observed also in other test including APTT, ACT but not anti- Xa [16]. The INR effect resides after 12 h which means after that period INR measurement are reliable. Clinicians should be aware of this effect to plan measurements in timely manner to avoid misinterpretation of lab results.

## Conclusions

In conclusion, our experience indicates that oritavancin is a valuable option to shorten and simplify the treatment of LVAD-related infections caused by Gram-positive bacteria, without compromising efficacy. This agent’s unique pharmacological profile and antibiofilm activity give it theoretical and practical advantages in this setting. Future studies are needed to determine the optimal timing of oritavancin initiation, dosing intervals for repeated use, and to further characterize its safety profile in advanced heart failure patients on mechanical support. Additionally, comparative trials between oritavancin and other therapy strategies (including dalbavancin or combination regimens) are required. Oritavancin’s successful use in our LVAD patient cohort, combined with accumulating evidence from other long-acting agents, marks an important step toward improving the care and outcomes of patients with LVAD infection.

## Limitations

This study should be interpreted with consideration of several limitations. The retrospective, non-comparative, single-center design may limit the generalizability of this data. Oritavancin was introduced after pre-treatment with standard-of-care antimicrobials. Apart from two episodes, in the remaining episodes no persistent signs and symptoms of active infection were present. On the other hand, not all of them had PET-CT performed what could affect treatment effect assessment.

## Data Availability

All the data mentioned in this paper.

## Abbreviations

ABSSSI: Acute Bacterial Skin And Skin Structure Infections
CIED: Cardiac Implantable Electronic Device
DLI: Drive-Line Infection
LVAD: Left Ventricle Assist Device
MSSA: Methicillin-Susceptible* Staphylococcus Aureus*
MRSA: Methicillin-Resistant*Staphylococcus Aureus*
OPAT: Outpatient Parenteral Antibiotic Therapy
PET-CT: Positron Emission Tomography
VISA: Vancomycin-Intermediate Resistant *Staphylococcus Aureus*
VRSA: Vancomycin Resistant *Staphylococcus Aureus*
VRE: Vancomycin Resistant Enterococci (Vre)
